# Multiple Bee Stings and Acute Kidney Injury: A Case Report

**DOI:** 10.7759/cureus.66488

**Published:** 2024-08-09

**Authors:** Veena V, Hemant Mehta, Preeti Dhingra, Anil Ballani

**Affiliations:** 1 Department of Medicine, Lilavati Hospital and Research Centre, Mumbai, IND; 2 Department of Nephrology, Lilavati Hospital and Research Centre, Mumbai, IND; 3 Department of Otorhinolaryngology, Lilavati Hospital and Research Centre, Mumbai, IND

**Keywords:** retrieval of a dead honeybee from a living patient, recovery from aki after bee stings, pathophysiology of bee sting induced aki, conservative management of aki, bee sting induced aki case report, acute kidney injury

## Abstract

Acute kidney injury (AKI) is a common complication following multiple honey bee stings and usually presents after 24-48 hours following the incidence. The severity of AKI is related to the number of stings. A single sting can cause an allergic reaction, and as the stings increase, a higher amount of venom is inoculated, leading to systemic poisoning. Bee venom can have direct or indirect effects on the kidneys. AKI is a combination of toxic and ischemic acute tubular necrosis. Patients may require dialysis, and the usual renal recovery time is 4-120 days. The patient with multiple honey bee stings needs emergency medical treatment, sometimes in the ICU setting, with the aim of treating or preventing anaphylaxis reactions. A case of AKI due to multiple honey bee stings is presented, which is rare but a known occurrence. The patient survived with a recovery of renal function.

## Introduction

Bees play an important role in pollinating flowering plants. Honeybees produce honey which has medicinal properties, but are also famous for their ability to inflict painful stings. Bees (family Apidae) is a venomous arthropod belonging to the order Hymenoptera whose venom is responsible for 14% of cases of anaphylactic reactions. The highest cause of anaphylaxis is food antigens (33-34% incidence) and the second highest cause of anaphylaxis reaction is stings of bees and wasps [1]. Their attack is usually in response to them being disturbed and to defend their colonies.

One or few stings are usually well tolerated, and the venom injected in humans causes pain at the site of sting, erythema, and edema or allergic reaction. A higher number of stings cause organ damage, and 50-500 stings could be fatal [2]. The affected organs include the brain, lungs, muscles, hematology, and kidneys. Acute kidney injury (AKI) is a common, known complication following multiple honey bee stings. The severity of AKI is related to the number of stings.

The mechanism of bee venom-induced AKI was studied in rats by injecting bee venom into them [3]. It showed an early and significant reduction in glomerular filtration rate, due to decreased renal blood flow associated with hypotension. The renal histopathology showed acute tubular necrosis (ATN) or myoglobinuric tubular injury. Bee venom and phospholipase A2 have been detected in the urine and plasma of the patients who were stung by bees [4]. This group III secretory phospholipase A2 from bee venom (bee venom group III sPLA2) is a known major allergen of bee venom that causes anaphylactic shock [5]. Glomerulonephritis with an increased serum IgE level and a reduced complement level has also been described [6].

AKI is a treatable but potentially fatal condition. In the severest form, the patient presents with shock associated with multiple stings and multiorgan dysfunction. Clinical responses to a sting range from minor local inflammation with or without envenomation to anaphylaxis [7]. The management should be prompt and aggressive for better patient outcomes. Source control is an important aspect of management.

We encountered one such case of bee sting injury which had renal and neurological involvement. He was managed in the critical care setting initially and later in the wards and made an uneventful recovery. A few dead bees were recovered from his nostril, following him sneezing out one honey bee. The patient's verbal consent was obtained before publication.

## Case presentation

**Figure 1:**
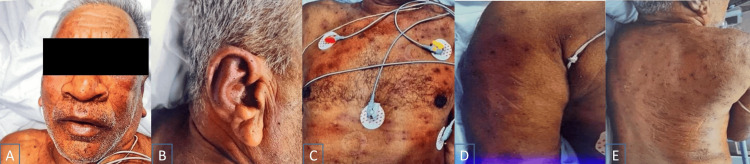
Multiple bee stings on the patient's (A) face, (B) lateral view of the face, (C) chest, (D) right arm, and (E) back Credits: Hemant Mehta

A 76-year-old villager was transferred to the tertiary care center with a history of stings by multiple honey bees on the previous day. He had a history of hypertension for four years but was otherwise healthy. On admission, he had altered sensorium, decreased urine output, and multiple skin lesions (Figure 1). Pulse was 90/min, regular, blood pressure was 110/70 mmHg, and he was breathing comfortably on room air with SpO_2 _of 98%. He was dehydrated and drowsy but arousable without any focal neurological deficit. His chest, precordium, and abdomen were normal on examination.

His initial investigations outside the hospital had shown hemoglobin of 15 gm/liter, platelet count of 302 K/mm^3^, and total leucocyte count of 24170/mm^3^, blood urea nitrogen (BUN) was 13 mg/dl, serum creatinine was 0.9 mg/d. On the day of admission at our hospital, BUN was 39 mg/dl and serum creatinine was 2.14 mg/dl. serum electrolytes showed sodium of 141.7 mEq/L, potassium of 5.3 mEq/L, chlorides of 106.9 mEq/L, and bicarbonate of 17.7 mEq/L. Urinalysis showed 1+ protein, but otherwise normal. A spot urine for the protein-creatinine ratio was 0.25 mg/mg.

**Figure 2:**
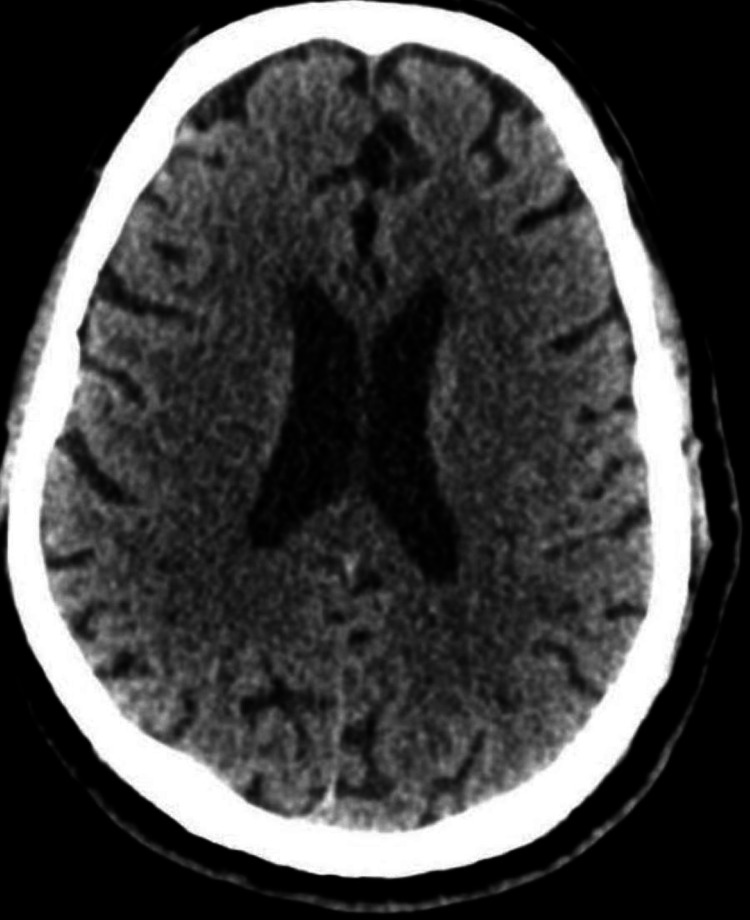
Plain CT scan of the brain with normal parenchyma and normal ventricles

**Figure 3:**
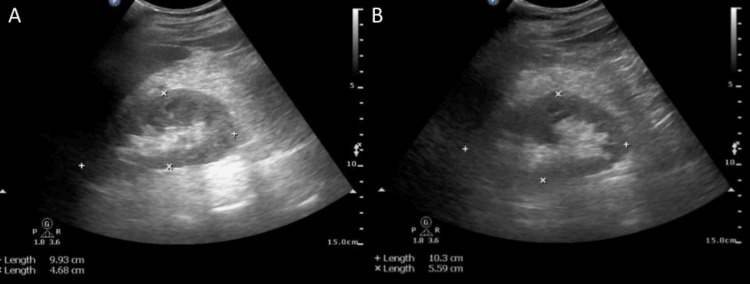
Ultrasonography of (A) right and (B) left kidneys, normal size with increased parenchymal echogenicity

A plain CT scan of the brain was normal (Figure 2). Ultrasonography of the abdomen and pelvis showed normal-sized kidneys with increased parenchymal echogenicity (Figure 3). It also showed a prominent common bile duct with mild intrahepatic biliary radical dilatation. He was started on IV fluids and IV antibiotics, ceftriaxone plus sulbactam, and clindamycin.

The next day, he was conscious and afebrile, hemodynamically stable. Hemoglobin had reduced to 12.3 gm/liter, platelets had reduced to 126 K/mm^3^, and leucocyte count was 25700/mm^3^. C-reactive protein was 31.8 mg/dl and S. complement 3 level was 121 mg/dl. He required an injection of furosemide to maintain his urine output. Over the next three days, leucocyte count showed a declining trend and S. creatinine rose up to 4.03 mg/dl, and repeat urinalysis showed protein 4+ with 60-70 RBCs. The blood culture sent on admission returned as negative. As the urine output improved, the diuretic was stopped.

**Figure 4:**
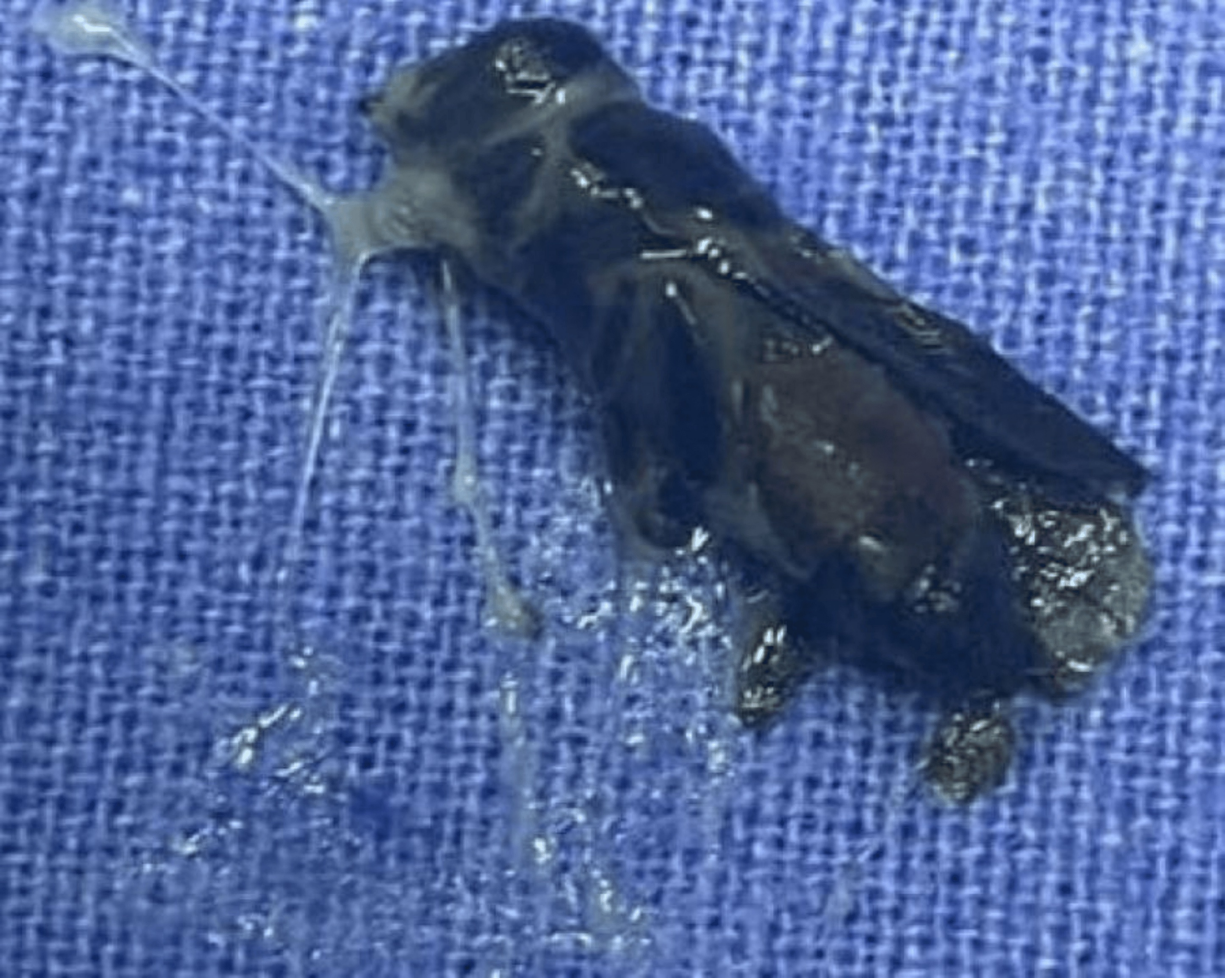
Actual photograph of the bee removed from the nostril Credits: Preeti Dhingra

On day 6 of admission, the patient complained of a tingling sensation in the ear and sneezed out honey bee from the nostril. An ENT opinion led to nasal endoscopy and removal of three dead honey bees from the right nostril (Figure 4) lying medial to medial turbinate and two from the left nostril lying near the posterior end of the inferior turbinate. An audiogram was normal. The patient was discharged on day 9 of admission and came for a follow-up a week later with a normal S. creatinine value. Table 1 displays laboratory results. 

**Table 1:** Laboratory parameters ^@^CRP: C-reactive protein; ^*^BUN: blood urea nitrogen; ^**^S: serum; ^***^RBC: red blood cells,^ #^ND: not done; ^^^: spot urine for protein - creatinine ratio ^$^Day -1 is one day prior to admission (at the outside facility); ^$$^Day 0 is the day of admission to our hospital, and then subsequent days; ^$$$^Day 1 is the next day of admission to our hospital, and so on till recovery on day 15

Laboratory parameters	Day -1^$^	Day 0^$$^	Day 1^$$$^	Day 2	Day 3	Day 4	Day 6	Day 15	Normal range
Hemoglobin	13.3	15	12.3	11.9	11.2	11.0	11.5	11.7	13-17 gm/dl
Leucocyte counts	12400	24170	25700	23200	20985	19640	15300	11460	4.0-10.0 (10^3^/cu.mm)
Platelets	326000	302000	126000	114000	106000	102000	136000	149000	150-410 (10^3^/cu.mm)
^@^CRP	^#^ND	31.8	ND	ND	ND	ND	ND	ND	<5 mg/L
*BUN	13	39	43	49	54	85	59	34	6-20 mg/dl
**S. creatinine	0.9	2.14	2.49	3.6	3.9	4.03	2.04	1.0	0.7-1.2 mg/dl
S. sodium	136	141.7	140.8	139.4	137.2	135.0	138.9	137.0	136-145 mEq/L
S. potassium	4.2	5.3	5.2	5.1	4.9	5.5	5.0	4.2	3.5-5.1 mEq/L
S. chlorides	99	106.9	102	109	114	112	105	106	98.0-107 mEq/L
S. bicarbonates	ND	17.7	15.0	14.1	13.2	11.5	18.1	23.9	20-29 mmol/L
Urinalysis	ND	^Protein 1+	ND	ND	ND	Protein 4+ ***RBC 60-70	ND	Protein absent	Protein absent

## Discussion

AKI following multiple honey bee stings usually presents after 24-48 hours following the incidence. A single sting can cause a local reaction and as the stings increase, a higher amount of venom is inoculated, leading to systemic poisoning. Bee venom is a complex mixture of proteins (phospholipase A2 and hyaluronidase enzymes) and peptides (melitin, apamin) which are the main poison components. Peptide 401, histamine, dopamine, and norepinephrine are the other poison components in the venom [1]. However, melitin is the main component that can be lethal. Besides, they can release histamine, bradykinin, serotonin, and prostaglandins. These being vasoactive, can cause hypotension and AKI.

**Figure 5:**
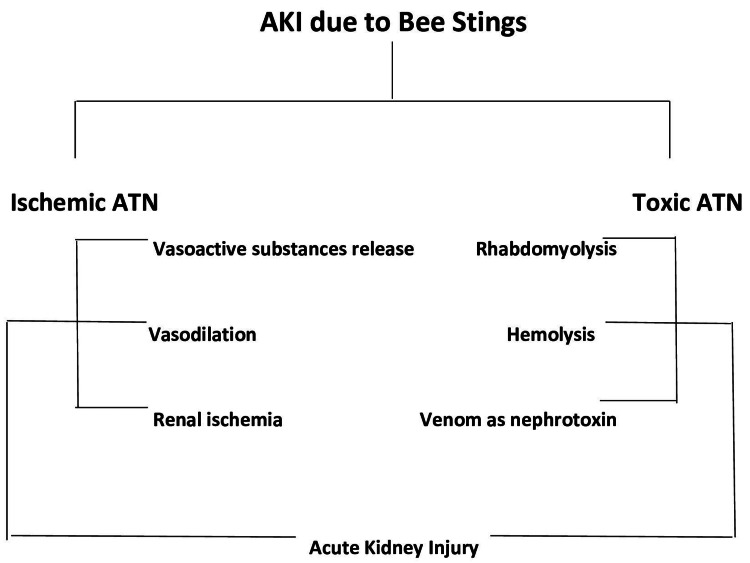
Pathophysiology of honey bee stings induced AKI Credits: Hemant Mehta AKI: acute kidney injury; ATN: acute tubular necrosis

Bee venom can have direct tubular toxicity on kidneys or indirectly it can affect the kidneys due to hemolysis, rhabdomyolysis, or shock. Our patient had no evidence of intravascular hemolysis or rhabdomyolysis on biochemical parameters (normal bilirubin and creatinine kinase levels). Renal lesions in the rats showing tubular damage and myoglobin deposition are attributed to renal vasoconstriction, even in the absence of hypotension or hemolysis [1]. It has also been observed that proximal renal tubules are affected by bee venom toxicity due to the reabsorption of toxins leading to metabolic activity and increased utilization of energy and enzymes [8]. As for the pathophysiology of honey bee sting-induced AKI, it is shown in Figure 5. It is a combination of toxic plus ischemic ATN. Toxic ATN is related to the toxins released by bee stings. The ischemic ATN would be anticipated in any situation that causes volume depletion (hypovolemia causing renal vasoconstriction), hypotension, and shock. Nitric oxide is an endothelial relaxing factor that also contributes to renal vasoconstriction [6].

Our patient had AKI and altered sensorium on presentation. The causes of renal and neurological involvement due to honey bee stings are different. Altered sensorium including coma has been known to be a part of the presenting feature of bees' stings [3], which could be due to systemic toxic reactions or rarely due to cerebral infarction or encephalomyelitis. Our patient was drowsy but arousable on admission. Since his sensorium improved and the CT scan of the brain without contrast was normal, we can presume the neurological status on admission to be due to a toxic reaction to the venom. Honey bee toxin can be fatal and the mortality due to honey bee stings is related to respiratory failure, anaphylactic shock, or myocardial infarction [9], and is estimated to be 15-25%. Our patient survived without any residual organ dysfunction.

A patient with multiple honey bee stings needs emergency medical treatment, sometimes in the ICU setting, with an aim to treat or prevent anaphylaxis reactions. Volume replacement, antihistamines, steroids, airway protection, and oxygen support are required as necessary. Prompt treatment of shock is crucial. AKI management is the same as for other situations of AKI. Hemodialysis may be indicated in some cases who have oligo-anuria, hyperkalemia, or metabolic acidosis. Our patient had borderline hyperkalemia (serum potassium of 5.3 mEq/L (normal up to 5.1)) and metabolic acidosis (venous bicarbonate level of 11.5 mmol/L, lowest level). Both of them responded to appropriate medical therapy of acidosis correction and supportive management of AKI and did not require hemodialysis.

We did not perform a kidney biopsy on our patient as renal function showed recovery. However, the most common renal histopathology in a series was ATN [3]. The required time of renal recovery is reported to range from 4 to 120 days [8], and our patient had renal function recovery by day 15, which is within the reported timeframe in the literature.

It is recommended that bees should be removed from the patient’s body as soon as possible, to reduce venom exposure time [4,6]. Venom inoculation is directly proportional to the sting-to-skin contact time. In our patient, source control could not be done immediately as there was no indication as to the bees lying inside the nostrils or sinuses. It was realized only after the patient sneezed out the bees. Immediate nasal endoscopy led to the complete clearance of bees from the patient’s body. Since the bees were dead by the time we realized that they were in the body, they must not have caused further venom exposure to the patient and hence patient had already shown signs of recovery while the bees were still in his body.

## Conclusions

A case of AKI due to multiple honey bee stings is presented, which is a known occurrence. The patient's AKI was managed conservatively, and source control was done. The patient survived with a recovery of renal function. It should be remembered that a bee sting is a medical emergency, and the first thing to suspect is anaphylaxis due to toxin, which should be treated accordingly with airway protection, fluid resuscitation, vasopressors as required, antihistaminics, and steroids if indicated. Source control is another important aspect of management. The management of AKI is as per standard guidelines and needs an aggressive approach as the situation is potentially fatal.
